# The secular trend of intelligence test scores: The Danish experience for young men born between 1940 and 2000

**DOI:** 10.1371/journal.pone.0261117

**Published:** 2021-12-09

**Authors:** Emilie R. Hegelund, Thomas W. Teasdale, Gunhild T. Okholm, Merete Osler, Thorkild I. A. Sørensen, Kaare Christensen, Erik L. Mortensen

**Affiliations:** 1 Department of Public Health, University of Copenhagen, Copenhagen, Denmark; 2 Department of Psychology, University of Copenhagen, Copenhagen, Denmark; 3 Center for Clinical Research and Prevention, Bispebjerg and Frederiksberg Hospitals, Frederiksberg, Denmark; 4 Department of Public Health, University of Southern Denmark, Odense, Denmark; University of Gothenburg: Goteborgs Universitet, SWEDEN

## Abstract

The present study investigated the Danish secular trend of intelligence test scores among young men born between 1940 and 2000, as well as the possible associations of birth cohort changes in family size, nutrition, education, and intelligence test score variability with the increasing secular trend. The study population included all men born from 1940 to 2000 who appeared before a draft board before 2020 (*N* = 1,556,770). At the mandatory draft board examination, the approximately 19-year-old men underwent a medical examination and an intelligence test. In the statistical analyses, the IQ mean and standard deviation (SD) were estimated separately for each of the included annual birth cohorts based on information from birth cohorts with available total intelligence test scores for all tested individuals (i.e. 1940–1958 and 1987–2000; the mean and SD were interpolated for the intermediate birth cohorts). Moreover, the possible associations with birth cohort changes in family size, height as a proxy for nutritional status, education, and IQ variability were investigated among those birth cohorts for whom a secular increase in intelligence test scores was found. The results showed that the estimated mean IQ score increased from a baseline set to 100 (SD: 15) among individuals born in 1940 to 108.9 (SD: 12.2) among individuals born in 1980, since when it has decreased. Focusing on the birth cohorts of 1940–1980, for whom a secular increase in intelligence test scores was found, birth cohort changes in family size, height, and education explained large proportions of the birth cohort variance in mean intelligence test scores, suggesting that these factors may be important contributors to the observed Flynn effect in Denmark.

## Introduction

Populations’ mean intelligence test scores have been rising since the beginning of the twentieth century. However, the phenomenon was first systematically documented by James Flynn in the U.S., and internationally, in the 1980s [1, 2]. For this reason, it is termed the *Flynn effect* [3]. Two large meta-analyses have found that the Flynn effect amounted to about three IQ points per decade during the past century [4, 5]. However, the trend has not been linear but rather seems to have been accelerating and decelerating and finally decreasing more recently [4]. In fact, a recent systematic review reports a reversed Flynn effect in seven European countries among individuals tested between 1975 and 2012 [6], and a data synthesis based on the aforementioned systematic review and another systematic review of a subset of secular losses likewise reports a reversed Flynn effect in 13 countries in Africa, Europe, and North and South America (study mid-years spanning from 1920.5 through 2007.5), suggesting that the reversed secular trend has strengthened in more recent years [7].

Possible explanations of the Flynn effect and its reversal have been numerous. Suggested explanations of the positive secular trend include changes in hybrid vigour (i.e. the mating of individuals from genetically dissimilar populations), family size, education, exposure to technology, test-taking behaviour, blood-lead levels, genomic imprinting, nutrition, pathogen stress, IQ variability, social multipliers, and life history speed [4]. However, current evidence points towards components of the life history speed theory [8], such as improvements in education and nutrition and a reduction in the prevalence of infectious diseases and other pathogens, as being the most important contributing factors [4]. Obviously, the negative trend may result from a diminishing influence of some of the aforementioned factors or an increasing influence of factors with negative impacts on intelligence.

In this study, the aim was to investigate the secular trend of intelligence test scores in Denmark. Previous Danish studies based on young male conscripts have suggested that intelligence test scores have increased with a decelerating rate between 1959 and 1998 (i.e. between the birth cohorts of 1940 and 1979), since then a slight fall and a subsequent stagnation have been observed [9–14]. However, the previous studies are mainly based on selected samples (e.g. men with specific birthdates and places of residence), samples from a few calendar years, or samples with only crudely categorized intelligence test scores available. Nonetheless, the previous studies have suggested that improvements in the population’s educational level and larger intelligence test score gains among the least able resulting in less IQ variability are important contributors to the last century’s secular increase in intelligence test scores, although other possible explanations remain unexplored. Only the most recent study has had the possibility to include the entire Danish population of men examined between 2006 and 2019 (closely corresponding to the birth cohorts of 1987–2000) and explore possible explanations of the secular decline in intelligence test scores [9]. This study found that neither genetic selection favouring those with lower intelligence, accumulation of deleterious mutations due to rising ages at parenthood, nor replacement migration as a consequence of migrants with higher fertility and lower intelligence moving to countries with lower fertility and higher intelligence seem to have contributed to the observed secular decline, but that changes in test format (from paper-and-pencil to computerized) and increasing sample selection are the most important contributors. However, there is a need for similarly thorough investigations of the secular trend before this study period to explore possible explanations of the secular increase in intelligence test scores as only the influences of changes in the population’s educational level and IQ variability formerly have been investigated.

Therefore, the present Danish study investigated the secular trend in intelligence test scores among young men born between 1940 and 2000 and investigated the associations with changes in family size, height as a proxy for nutritional status, education, and IQ variability among those birth cohorts where an increase in intelligence test scores was observed.

## Materials and methods

### Study population

A register-based cohort study was conducted of all Danish men born from 1940 through 2000 and appearing before a draft board before 2020 (*N* = 1,556,770).

All Danish men are required to appear before a draft board when they turn 18 years old in order to evaluate their suitability for military service. The men can either be declared fully eligible, limitedly eligible, or ineligible, based on the results of an intelligence test and a medical examination. About half of those declared ineligible never appear before a draft board as they are exempted from military service due to documentation of health issues forwarded in advance to the draft board. For the birth cohorts of 1940 through 1959, this involved around 5–9% of each birth cohort with a slight increase observed over time among those born in the 1950s. However, this number has increased substantially in the last decade from 13% of the men born in 1987 to around 20% of the men in the youngest birth cohorts.

According to Danish legislation, no ethical approval is needed for register-based studies, but the present study is covered by the approval of the Danish Data Protection Agency.

### Variables

#### Intelligence

Intelligence was measured by the intelligence test Børge Priens Prøve (the BPP). The BPP was introduced for draft board assessment purposes in 1957 and has been in continual use since then. It comprises four subtests measuring logical, verbal, numerical and spatial ability. Each subtest includes approximately 20 items, none of which are multiple-choice. The resulting test score is given by the total number of correct answers summed across the four subtests (range: 0–78). The items in the subtests have remained unchanged to the present day, although their presentation changed in 2010 from a paper-and-pencil booklet to a computer-administered format. The subtests have been shown to have a strong ‘g’ component [15] and the total score correlates highly with the widely-used Wechsler Adult Intelligence Test [16] and Raven’s Progressive Matrices [17]. However, the intelligence test is essentially a test of fluid intelligence [18, 19]. More detail on the BPP is presented elsewhere [17].

Information on intelligence test scores was available from four conscription databases covering various birth cohorts: the Danish Conscription Database (~1940–1958), the Danish National Archive’s database (~1969–1986), the Danish Defence Personnel Organisation’s database (~1976–1986), and the Conscription Registry (~1987-). However, in the Danish National Archive’s database, the raw total score had been condensed into five categories and in the Danish Defence Personnel Organisation’s database, only those declared fully and limitedly eligible were included. In other words, only the birth cohorts of 1940–1958 and 1987–2000 had total intelligence test scores available for all tested conscripts.

#### Contributors to the secular increase in intelligence test scores

Family size, height as a proxy for nutritional status, and education were measured on both birth cohort and individual levels. More specifically, family size was measured on birth cohort level using information about the Danish population’s fertility rate (that is, the number of live births per 1,000 women of childbearing age) in a given calendar year and on individual level as the individual’s number of siblings, which were available from Statistics Denmark’s publicly available website (www.statbank.dk) and their population registers, respectively. Height (in centimetres) was used as a proxy for the sample’s nutritional status as it has been found to be a useful indicator of variation in cumulative net nutrition, particularly the intake of high-quality proteins [20]. It was measured as part of the medical examination during the draft board examination. The mean height of all men belonging to a specific birth cohort was used in the analyses on cohort level, while the individual’s height was used in the analyses on individual level. Education was measured as the number of years in education, which were reported at the draft board examination. The mean educational attainment of all men belonging to a specific birth cohort was used in the analyses on cohort level, while the individual’s educational attainment was used in the analyses on individual level.

### Statistical methods

The main analysis investigated the secular trend of intelligence test scores among individuals born between 1940 and 2000 by separately estimating the IQ mean and standard deviation (SD) for each birth cohort based on two linear regression models with linear, quadratic, and cubic birth year terms as independent variables. Since only the birth cohorts of 1940–1958 and 1987–2000 had intelligence test scores for all tested conscripts, only these birth cohorts’ means and SDs were included in the linear regression models. The two models explained 96.8% and 99.0% of the variance in the birth cohorts’ IQ mean and SD, respectively, and they were used to interpolate the mean and SD of the incomplete birth cohorts such that the secular trend of intelligence test scores could be illustrated during the entire study period. The birth cohort of 1940 was chosen as the baseline and its intelligence test score distribution was standardized to have a mean of 100 and an SD of 15. The subsequent birth cohorts’ intelligence test scores were scaled against this baseline.

Subsequently, the possible associations with changes in family size, height, education, and IQ variability were investigated among those birth cohorts for whom a secular increase in intelligence test score was found (that is, the birth cohorts of 1940–1980). First, secular trends in mean family size, height, education, and IQ variability were explored by calculating annual means and standard deviations. Second, the correlations of the birth cohorts’ average family size, height, and education with annual mean intelligence test scores were calculated based on the empirical observations and the same associations were estimated by use of three separate linear regression models as well as one combined model including all factors. Third, the combined model was expanded by including birth cohort as a further explanatory factor. Finally, the abovementioned analyses were repeated using individual-level data to compare the investigated associations at cohort- and individual levels as the evaluation of potential contributing factors to a secular trend should be based on the total accumulated evidence considering the associations at both cohort- and individual levels. That is, the associations of the individual’s number of siblings, height, and education with its intelligence test score were calculated. The possible associations with changes in family size, height, education, and IQ variability were only investigated among those birth cohorts for whom a secular increase in intelligence test score was found as we in a previous study have looked at the possible explanations of the secular decline.

All statistical analyses were carried out using Stata version 16.1.

## Results

### Secular trend of intelligence test scores among the birth cohorts of 1940–2000

**Fig 1:**
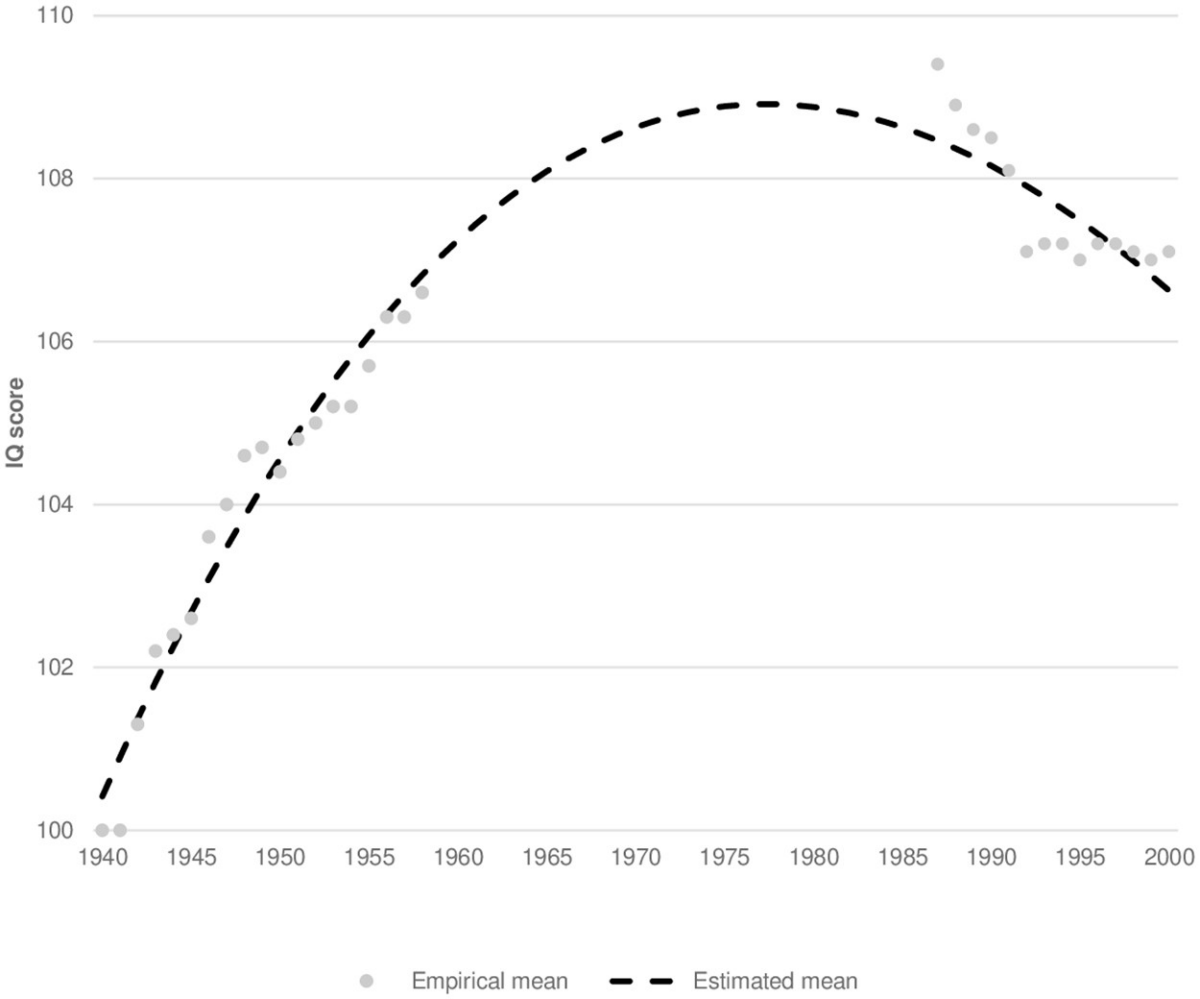
Mean intelligence test score according to annual birth cohort.

The estimated mean intelligence test scores of the birth cohorts of 1940 to 2000 showed a curvilinear relation (Fig 1 and S1 Table). Interpolating the mean for those birth cohorts that did not have total intelligence test scores available for all tested conscripts suggested that the IQ mean increased from 100 (SD: 15) for those born in 1940 to 108.9 (SD: 12.2) for those born in 1980 since when it has been decreasing. More specifically, we estimated an average increase of 3.8 IQ points for the birth cohorts of 1940–49, 2.5 IQ points for the birth cohorts of 1950–59, 1.3 IQ points for the birth cohorts of 1960–69, and 0.3 IQ points for the birth cohorts of 1970–79. Hereafter, we estimated an average decrease of 0.6 IQ points for the birth cohorts of 1980–89 and 1.3 IQ points for the birth cohorts of 1990–99. However, looking at the empirical data, it is clear that the estimated change for the last birth decade conceals a true decline of 0.4 IQ points from those born in 1990 to 1991, a sudden drop of 1.0 IQ points from those born in 1991 to 1992 (coinciding with the change in the format of the intelligence test from a paper-and-pencil booklet to a computer-administered format), and a following stagnation. Yet the interpolated mean intelligence test scores across birth cohorts looked exactly the same irrespective of whether individuals born after 1992 were excluded from the linear regression model or not. The standard deviation declined steadily during the study period from 15.0 for individuals born in 1940 to 10.0 for individuals born in 2000. Furthermore, the symmetrical IQ distribution observed at the beginning of the study period turned into a left-skewed one concurrently with the increase in mean intelligence test scores.

### Possible explanations of the increasing secular trend among the birth cohorts of 1940–1980

Focusing on the birth cohorts of 1940 to 1980, for whom a secular increase in intelligence test scores was estimated, the mean family size was found to decrease across birth cohorts whereas the height and educational level increased. More specifically, the Danish population’s fertility rate first increased from 2.2 children per woman in 1940 to 3.0 children per woman in 1946, after which it declined to 1.5 children per woman in 1980. Meanwhile, the mean height increased from 175 cm among those born in 1940 to 180 cm among those born in 1980 and the median education level increased from 9 years among those born in 1940 to 11 years among those born in 1980. The cohort-level correlations between the potential explanatory factors and intelligence test scores were r_family size_: -0.75, r_height_: 0.97, and r_education_: 0.99. Moreover, the IQ variability declined strongly across birth cohorts. Thus, the birth cohort of 1940 had a standard deviation of 15, whereas the birth cohort of 1980 had a standard deviation of 12.2. The correlation between the birth cohorts’ mean and variance in intelligence test scores was *r* = -0.98 (*p*<0.001).

The observed changes in family size, height, and education separately explained a large amount of the variance in the birth cohorts’ annual mean intelligence test scores (Table 1). Collectively, the three factors explained 99.6% of the variance in the birth cohorts’ mean intelligence test scores (S2 Table).

**Table 1:** Explained variance in mean intelligence test scores among the birth cohorts of 1940–1980.

Parameter	Cohort-level associations		Individual-level associations	
	*r*	R^2^	*r*	R^2^
Family size	-0.75	0.726	-0.06	0.014
Height	0.97	0.990	0.26	0.067
Education	0.99	0.994	0.66	0.487

Interestingly, there was still a significant association between birth cohort and intelligence test scores when birth cohort changes in family size, height, and education had been accounted for (*p*<0.001). However, the residual influence of birth cohort explained less than 1% of the variance in mean intelligence test scores.

All the above statistical analyses were replicated in sensitivity analyses solely based on data from the birth cohorts of 1940 to 1958 who had total intelligence test scores available for all tested conscripts (S3 Table). The findings were generally consistent, except that the cohort-level correlation between family size and mean intelligence test scores was positive and family size explained a little less of the variance in mean intelligence test scores.

## Discussion

### Main findings

The estimated mean IQ score of the Danish male birth cohorts of 1940 through 2000 increased from a baseline set to 100 (SD: 15) for those born in 1940 to 108.9 (SD: 12.2) for those born in 1980 followed by a secular decline for two decades. However, looking at the empirical data, it is clear that the estimated decline during the last decade conceals a true decline of 0.4 IQ points from those born in 1990 to 1991, a sudden drop of 1.0 IQ points from those born in 1991 to 1992 (coinciding with the change in the format of the intelligence test), and a following stagnation. Focusing on the birth cohorts of 1940–1980, for whom a secular increase in intelligence test scores was found, birth cohort changes in family size, height, and education all explained a large proportion of the variance in the birth cohorts’ mean intelligence test scores. Moreover, the IQ variance was found to decrease strongly across birth cohorts.

### Comparison with the existing literature

The estimated curvilinear development in mean intelligence test scores of the Danish male birth cohorts born between 1940 and 2000 is supported by previous Danish studies based on somewhat selected samples of our study population and also partly by a global meta-analysis of studies covering an entire century reporting decreasing intelligence test score gains in more recent decades [4, 9–14]. Moreover, the curvilinear development seems to be virtually identical with changes in spatial ability observed in German-speaking countries during the period of 1977 to 2014 [21]. Yet, as noted above, the empirical data suggest that the estimated secular decline for the last decade conceals a true decline of 0.4 IQ points from those born in 1990 to 1991, a sudden drop of 1.0 IQ points from those born in 1991 to 1992, and a following stagnation. However, in a previous study, we found that changes in sample composition during this decade may have inflated the mean intelligence test scores as the proportion of exempted increased and this group generally seemed to be worse off with regard to characteristics that were all correlated with intelligence [9]. In other words, if a larger proportion of individuals with low intelligence got exempted from testing, the observed stagnation in mean intelligence test scores might conceal a true secular decline.

Focusing on the birth cohorts of 1940 to 1980, for whom a secular increase in intelligence test scores was estimated, we observed that the population’s mean family size simultaneously decreased, whereas height as a proxy for the population’s nutritional status and educational level increased. In other words, the secular increase in intelligence test scores coincided with changes in the three potential explanatory factors, manifesting itself in the statistical models’ large amounts of explained variance. However, it is important to note that this correlational study in itself cannot provide evidence of the causal influences of the potential explanatory factors, and the reported amounts of explained variance should not be interpreted as causal influences, but rather as indications of whether the three factors may be related to the secular trend in intelligence test scores. We assume that if an explanatory factor has a substantial causal influence on the observed secular increase in intelligence test scores, it must show a high correlation with intelligence at the cohort level. This is indeed the case for the explanatory factors in this study. However, many factors related to the population’s life circumstances change over time and, presumably, changes in some factors may correlate with changes in the birth cohorts’ mean intelligence test scores even though the two phenomena are completely unrelated. This means that the high correlations can both reflect causal associations between the explanatory factors and mean intelligence test scores or confounding from factors influencing the explanatory factors and mean intelligence test scores. Yet if we are dealing with causal associations it should also be possible to identify significant correlations at the individual level, which is indeed the case here. These correlations suggest that education is much more strongly related to intelligence than the two other factors, but this may to some extent reflect reciprocal causation as intelligence influences the individual’s educational attainment. Moreover, it is important to note that the magnitude of the correlations depends on the precision of measurement of the explanatory factors and the between- and within-cohort variance. For example, height may be a good proxy measure for the population’s nutritional status at the cohort level, but because of its high heritability, it is not precise at the individual level [20] and there may be limited variance in critical nutritional factors in the Danish population. In other words, the moderate individual-level correlation between height and intelligence does not exclude a possible causal influence of the Danish population’s nutritional status on mean intelligence test scores across birth cohorts. Consequently, the evaluation of potential contributing factors to an observed secular trend should be based on the total accumulated evidence across studies examining the associations at both cohort- and individual levels.

Consistent with our study findings, it has been suggested that populations’ decreasing family sizes have led to increasing mean intelligence test scores due to the influences of birth order and its associated side effects [22]. As intelligence test scores have been found to decrease with increasing birth order because parents have less time and resources to spend on each child, populations’ decreasing family sizes may lead to increasing mean intelligence test scores simply due to the larger proportion of firstborn children. However, a larger proportion of firstborn children will contribute positively to the intellectual environment of everyone in the birth cohort and for this reason, the birth cohort’s mean intelligence test score may increase further. This may explain why the observed association between family size and intelligence was considerably larger in our cohort-level analyses than in our individual-level analyses. Yet, the reverse association between family size and intelligence is not unambiguously supported. Thus, a meta-analysis found that fertility rate was positively related to intelligence test score gains [4]. The inconsistent findings may be due to contextual differences in the influence of family size or due to the meta-analysis’ crude analysis estimating an average influence of family size across time periods with both increasing and decreasing family sizes. As is evident in our sensitivity analysis, the timing of the analysis of the contributing factors may have a large impact on the findings.

Improved prenatal and postnatal nutrition may also have contributed to the secular increase in intelligence test scores as nutrition, particularly high-quality protein, influences the makeup of the human body, such as body height, head circumference, and intelligence [20, 23–25] and we observed simultaneous increases in body height and intelligence. This observation is in line with a previous study, which also observed a notable increase in Danish men’s body height among those born between 1940 and 1980 [26]. Moreover, the increases in body height and intelligence occurred at a time during which both the quantity and quality of the Danish population’s nutrition, particularly its meat consumption, increased markedly as a consequence of considerable improvements in living standards [27]. While suboptimal prenatal and postnatal nutrition has been found to reduce body height, head circumference, and intelligence, improvements in nutrition on the other hand have a positive influence on the human body, including the development and the functioning of the brain [23, 24]. However, the beneficial influence of nutrition may, by and large, have reached its limit during the study period, which would also explain the decreasing association between height and intelligence [28] and the decreasing intelligence test score gains over time. Moreover, given that malnourished individuals are likely to benefit more from improved nutrition, our observation of a decreasing intelligence test score variance would be expected.

Danish studies have suggested that the observed secular trend of intelligence test scores has mirrored the country’s educational changes [10–12]. Thus, it has been shown that the secular increase was strongest among those with low intelligence test scores and that this coincided with an increase in the population’s minimum educational level and increasing resources being allocated to remedial and supplementary education. Like the previous Danish studies, we also observed that the secular increase in intelligence test scores was strongest among those with low intelligence (S1 Fig). This may be explained by the population’s increasing educational level as we previously have shown that the positive influence of education on intelligence test scores is strongest among those with low intelligence in childhood [29]. Moreover, we have previously shown that the positive influence on intelligence depends on the educational level and that it starts to decline after upper-secondary school [29], which supports our observation of smaller increases in the population’s mean intelligence test score over time. Generally, the positive influence of education on intelligence test scores may be due to various mechanisms, such as the teaching of information directly relevant to intelligence tests, the teaching of thinking styles, and the inculcating of concentration and self-control that may improve test performance [30, 31]. The influence of education on the secular trend in intelligence test scores is supported by a wide range of evidence, including findings of a substantial global increase in crystallized intelligence and stronger intelligence test score gains among adults than children [4]. However, while education is primarily expected to influence crystallized intelligence, the intelligence test used in our study is essentially a test of fluid intelligence [18, 19] and the observed high correlations between education and intelligence are therefore remarkable.

As noted above, our observation of a decreasing intelligence test score variance seems to reflect a stronger secular increase in intelligence test scores among those with low intelligence [11, 12]. This reduced variability may, as suggested, both be explained by improved nutrition primarily benefiting malnourished individuals [32, 33] and educational changes mainly improving the abilities of the least able [34]. In a Danish context, both of these explanations seem reasonable since the decreasing intelligence test score variance coincided with an increasing high-quality protein intake, as well as an increase in the population’s minimum educational level and increasing resources being allocated to remedial and supplementary education. Thus, our findings support the hypothesis that both nutrition and education may be important contributing factors to the secular increase in mean intelligence test scores and the decreasing variance. Overall, our study findings thus lend support to the ‘life history speed’ theory [8], suggesting that the society’s reduced perceived mortality threat due to improved nutrition and less pathogen stress results in a decreased life history speed over time (which is related to factors such as reduced family size and better education) and allow for investment into cognitive ability maturation, which may ultimately explain the observed secular increase.

### Strengths and limitations

The major strength of the present study is its large study population, including >1 million Danish men born in 1940–1958 and 1987–2000 and appearing before a draft board until 2020, which has made it possible to analyse the secular trend of intelligence test scores in the same population over sixty years. Other strengths include the use of information collected at the draft board examination, including, inter alia, educational level and height measured on-site. The use of the same well-validated intelligence test can likewise be considered a strength. Although the use of the same test also makes obsolescence of test items possible, this is less of a concern, given that the test items involve abstract reasoning, which is less susceptible to obsolescence.

However, since only total intelligence test scores were available, it was not possible to evaluate whether our intelligence construct changed over time. Likewise, it was not possible to evaluate whether the secular trend of intelligence test scores was homogeneous across subtests or if the trend depended on the subtests’ g-loadings which might have given us hints about which factors contributed to the observed secular increase. Another limitation is that only the birth cohorts born in 1940–1958 and 1987–2000 had total intelligence test scores available for all tested conscripts and, therefore, their intelligence test score distributions were used to interpolate the secular trend during the entire study period. The regression model used to interpolate the missing birth cohort values explained a large proportion of the variance in mean intelligence test scores, but as previously written the estimated mean differed from the empirical mean observed during the last birth decade. Also, the investigation of the possible contributors to the secular increase in intelligence test scores may have been influenced by the specific birth cohorts with available intelligence test scores; for instance, the sensitivity analysis suggested that the influence of family size differed when considering only the birth cohorts of 1940–1958 versus all birth cohorts born until 1980 with available intelligence test scores (i.e. 1940–1959 and 1976–1980). Moreover, there has been a rising proportion of conscripts getting exempted from the draft board examinations over time and a previous study has suggested that this may, artificially, have inflated the population mean among the youngest birth cohorts as the group of exempted individuals generally seems to be worse off with regard to various characteristics correlated with intelligence [9]. Finally, it should be mentioned that there is no evidence of major sex differences in general intelligence test scores [35], but the observed secular trend may be limited to young people born in the same time and place as our study population since previous studies have suggested that the secular trend of intelligence test scores vary across age and countries [4, 36]. However, the explanatory factors contributing to the secular trend may be more generalizable although the magnitude of their impact likewise may be limited to societies similar to the Danish society during the study period.

## Conclusions

This Danish population-based study found that the estimated mean IQ score increased from a baseline set to 100 (SD: 15) among men born in 1940 to 108.9 (SD: 12.2) among men born in 1980, whereafter it decreased. This finding was based on a regression model of the mean intelligence test scores of the birth cohorts of men born in 1940–1958 and 1987–2000. Focusing on those birth cohorts for whom a secular increase in intelligence test scores was found, simultaneous reductions in family size and rises in height (as a proxy for nutritional status) and education were found to explain large proportions of the variance in the birth cohorts’ mean intelligence test scores, suggesting that these three factors may all have contributed to the secular increase of intelligence test scores. The simultaneous reduction in IQ variance, due to a stronger secular increase among those with low intelligence, lends further support to the potential importance of birth cohort changes in nutrition and educational attainment. In conclusion, the study suggests that components of the ‘life history speed’ theory are important contributors to the Flynn effect observed across four decades in Denmark.

## Supporting information

S1 Fig Mean intelligence test score according to birth cohort among individuals in selected percentiles (P10, P25, P75, and P90) of the intelligence test score distribution. The 1959 birth cohort is not complete and the 1976–86 birth cohorts only include men declared fully and limitedly eligible for military service.
(DOCX)

S1 Table Observed mean intelligence test score and standard deviation according to birth cohort. (DOCX)

S2 Table Associations of the birth cohorts’ average family size, height, and education with mean intelligence test scores among individuals born from 1940 to 1980. (DOCX)

S3 Table Explained variance in mean intelligence test scores among the birth cohorts of 1940–1958. (DOCX)
