# Supplementary material for: The secular trend of intelligence test scores: The Danish experience for young men born between 1940 and 2000
Source: PLoS One. 2021 Dec 9;16(12):e0261117. doi: 10.1371/journal.pone.0261117 (PMC8659667; doi:10.1371/journal.pone.0261117)
Supplement: S1 Fig — The 1959 birth cohort is not complete and the 1976–86 birth cohorts only include men declared fully and limitedly eligible for military service. (DOCX) [file pone.0261117.s001.docx]

**S1 Fig. Mean intelligence test score according to birth cohort among individuals in selected percentiles (P10, P25, P75, and P90) of the intelligence test score distribution.** The 1959 birth cohort is not complete and the 1976-86 birth cohorts only include men declared fully and limitedly eligible for military service.
