# Supplementary material for: The secular trend of intelligence test scores: The Danish experience for young men born between 1940 and 2000
Source: PLoS One. 2021 Dec 9;16(12):e0261117. doi: 10.1371/journal.pone.0261117 (PMC8659667; doi:10.1371/journal.pone.0261117)
Supplement: S1 Table — (DOCX) [file pone.0261117.s002.docx]

**S1 Table.** **Observed mean intelligence test score and standard deviation according to birth cohort.**

| Birth cohort | *N* | *M* (*SD*) | Skewness | Kurtosis |
| --- | --- | --- | --- | --- |
| 1940 | 28,181 | 100.0 (15.0) | 0.01 | 2.45 |
| 1941 | 28,410 | 100.0 (15.1) | 0.05 | 2.46 |
| 1942 | 32,920 | 101.3 (15.0) | -0.03 | 2.44 |
| 1943 | 35,876 | 102.2 (15.0) | -0.06 | 2.44 |
| 1944 | 37,956 | 102.4 (14.9) | -0.07 | 2.43 |
| 1945 | 39,984 | 102.6 (14.8) | -0.07 | 2.41 |
| 1946 | 40,735 | 103.6 (14.5) | -0.10 | 2.44 |
| 1947 | 39,817 | 104.0 (14.1) | -0.10 | 2.51 |
| 1948 | 35,869 | 104.6 (13.9) | -0.14 | 2.55 |
| 1949 | 30,983 | 104.7 (13.8) | -0.15 | 2.55 |
| 1950 | 35,926 | 104.4 (13.9) | -0.16 | 2.56 |
| 1951 | 33,968 | 104.8 (13.9) | -0.20 | 2.58 |
| 1952 | 31,287 | 105.0 (13.9) | -0.22 | 2.65 |
| 1953 | 31,778 | 105.2 (13.7) | -0.25 | 2.68 |
| 1954 | 30,672 | 105.2 (13.5) | -0.26 | 2.68 |
| 1955 | 32,739 | 105.7 (13.3) | -0.27 | 2.68 |
| 1956 | 32,750 | 106.3 (13.2) | -0.30 | 2.76 |
| 1957 | 29,053 | 106.3 (13.1) | -0.31 | 2.75 |
| 1958 | 29,089 | 106.6 (13.0) | -0.31 | 2.74 |
| 1959^a^ | 18,914 | 107.1 (13.0) | -0.31 | 2.72 |
| … |  |  |  |  |
| 1976^b^ | 30,001 | 111.3 (10.3) | -0.11 | 2.72 |
| 1977^b^ | 28,236 | 111.8 (10.2) | -0.12 | 2.72 |
| 1978^b^ | 28,259 | 112.1 (9.8) | -0.05 | 2.72 |
| 1979^b^ | 27,193 | 112.1 (9.9) | -0.00 | 2.66 |
| 1980^b^ | 26,384 | 112.2 (9.8) | -0.05 | 2.75 |
| 1981^b^ | 24,393 | 111.8 (10.0) | -0.05 | 2.95 |
| 1982^b^ | 24,171 | 111.8 (10.0) | -0.03 | 2.74 |
| 1983^b^ | 23,284 | 111.8 (10.0) | -0.09 | 2.94 |
| 1984^b^ | 23,640 | 111.4 (10.1) | -0.14 | 3.03 |
| 1985^b^ | 23,806 | 111.0 (10.4) | -0.20 | 3.20 |
| 1986^b^ | 23,674 | 110.4 (10.8) | -0.29 | 3.27 |
| 1987 | 24,711 | 109.4 (11.7) | -0.43 | 3.29 |
| 1988 | 26,757 | 108.9 (11.9) | -0.45 | 3.27 |
| 1989 | 27,481 | 108.6 (11.7) | -0.50 | 3.36 |
| 1990 | 28,345 | 108.5 (11.6) | -0.45 | 3.29 |
| 1991 | 28,606 | 108.1 (11.4) | -0.42 | 3.27 |
| 1992 | 29,492 | 107.1 (11.3) | -0.40 | 3.30 |
| 1993 | 28,777 | 107.2 (11.1) | -0.42 | 3.34 |
| 1994 | 29,255 | 107.2 (10.8) | -0.37 | 3.32 |
| 1995 | 28,955 | 107.0 (10.6) | -0.34 | 3.25 |
| 1996 | 27,705 | 107.2 (10.5) | -0.38 | 3.42 |
| 1997 | 27,616 | 107.2 (10.5) | -0.35 | 3.36 |
| 1998 | 26,242 | 107.1 (10.5) | -0.37 | 3.31 |
| 1999 | 24,424 | 107.0 (10.2) | -0.34 | 3.46 |
| 2000 | 23,585 | 107.1 (10.2) | -0.34 | 3.40 |

^a^The 1959 birth cohort is not complete and for this reason, its mean and SD are not included in the regression models.

^b^The 1976-86 birth cohorts only included men declared eligible and limitedly eligible for military service. For this reason, their means and SDs are not included in the regression models.
