# Supplementary material for: The secular trend of intelligence test scores: The Danish experience for young men born between 1940 and 2000
Source: PLoS One. 2021 Dec 9;16(12):e0261117. doi: 10.1371/journal.pone.0261117 (PMC8659667; doi:10.1371/journal.pone.0261117)
Supplement: S2 Table — (DOCX) [file pone.0261117.s003.docx]

**S2 Table.** **Associations of the birth cohorts’ average family size, height, and education with mean intelligence test scores among individuals born from 1940 to 1980.**

| Parameter | Estimate | SE | *β* | *p* |
| --- | --- | --- | --- | --- |
| Intercept | 98.96 | 0.01 | - | <0.001 |
| Family size | 0.01 | 2.12x10^-5^ | 0.66 | <0.001 |
| Family size^2^ | -8.73x10^-6^ | 2.52x10^-8^ | -1.55 | <0.001 |
| Family size^3^ | 3.37x10^-9^ | 9.20x10^-12^ | 0.87 | <0.001 |
| Height | 1.23 | 0.01 | 0.61 | <0.001 |
| Height^2^ | -0.76 | 1.37x10^-3^ | -2.42 | <0.001 |
| Height^3^ | 0.12 | 1.93x10^-4^ | 0.66 | <0.001 |
| Education | 14.38 | 0.03 | 2.77 | <0.001 |
| Education^2^ | -9.99 | 0.03 | -4.64 | <0.001 |
| Education^3^ | 2.39 | 0.01 | 2.47 | <0.001 |
| *F* | 99,999.00 |  |  | <0.001 |
| *R^2^* | 0.996 |  |  |  |
