# Supplementary material for: The secular trend of intelligence test scores: The Danish experience for young men born between 1940 and 2000
Source: PLoS One. 2021 Dec 9;16(12):e0261117. doi: 10.1371/journal.pone.0261117 (PMC8659667; doi:10.1371/journal.pone.0261117)
Supplement: S3 Table — (DOCX) [file pone.0261117.s004.docx]

**S3 Table.** **Explained variance in mean intelligence test scores among the birth cohorts of 1940-1958.**

| Parameter | Cohort-level associations | |  | Individual-level associations | |
| --- | --- | --- | --- | --- | --- |
|  | *r* | R^2^ |  | *r* | R^2^ |
| Family size | 0.13 | 0.563 |  | -0.08 | 0.010 |
| Height | 0.97 | 0.977 |  | 0.25 | 0.061 |
| Education | 0.96 | 0.983 |  | 0.68 | 0.492 |
